# Guideline of guidelines: management of small testicular masses

**DOI:** 10.1111/bju.70131

**Published:** 2025-12-24

**Authors:** Marco Tozzi, Giuseppe Fallara, Matteo Ferro, Francesco Chierigo, Roberto Bianchi, Hussain M. Alnajjar, Karl H. Pang, Asif Muneer

**Affiliations:** ^1^ Unit of Urology ASST Santi Paolo and Carlo Milan Italy; ^2^ Department of Health Science La Statale University of Milan Milan Italy; ^3^ Department of Andrology University College London Hospitals NHS Foundation Trust London UK; ^4^ Division of Surgery and Interventional Science University College London London UK; ^5^ Department of Urology Chelsea and Westminster Hospital NHS Foundation Trust London UK; ^6^ National Institute for Health and Care Research UCLH Biomedical Research Centre London UK

**Keywords:** small testicular mass, testis‐sparing surgery, testicular surveillance, testis biopsy, orchidectomy, frozen section, ultrasound

## Abstract

**Objective:**

To compare and summarise the most up to date international guidelines and major recommendations for the management of small testicular masses (STMs).

**Methods:**

A systematic search was conducted in PubMed, EMBASE, Scopus, Google Scholar, and the Cochrane Library up to 1 November 2024. The latest editions of five international guidelines were included in the review: the European Association of Urology 2025; the National Comprehensive Cancer Network 2024; the American Urological Association 2023; the Canadian Urological Association 2022; and the European Society for Medical Oncology 2018. The Appraisal of Guidelines for Research and Evaluation II (AGREE II) tool was applied by two authors (M.T. and G.F.) to assess the quality of these guidelines.

**Results:**

Emerging evidence supports testis‐sparing surgery (TSS) as a viable option for managing STMs, showing acceptable oncological outcomes and preservation of gonadal function in select patients. The shared indications for TSS include indeterminate STMs identified on ultrasonography with negative tumour markers, particularly in the case of a solitary testis or of bilateral tumours. Where intra‐operative frozen section analysis is available, and confirms a benign lesion, a radical orchidectomy could be avoided.

**Conclusion:**

Consensus across guidelines favours TSS in suitable cases, balancing oncological control with functional outcomes and informed patient decisions.

**Abbreviations**
AGREE II: Appraisal of Guidelines for Research and Evaluation II
AS: active surveillance
CDUS: colour‐Doppler ultrasonography
CECT: contrast‐enhanced CT
CEUS: contrast‐enhanced ultrasonography
CUA: Canadian Urological Association
EAU: European Association of Urology
ESMO: European Society for Medical Oncology
FSE: frozen section examination
GCNIS: germ cell neoplasia *in situ*
LCT: Leydig cell tumour
microTESE: microdissection testicular sperm extraction
miRNA: microRNA
mpUS: multiparametric ultrasonography
NCCN: National Comprehensive Cancer Network
oncoTESE: oncological testicular sperm extraction
RO: radical orchidectomy
SE: sonoelastography
STM: small testicular mass
TC: testicular cancer
TSS: testis‐sparing surgery
US: ultrasonography

## Introduction

Testicular cancer (TC) is a rare cancer, accounting for ≈1% of all male cancers and ≈5% of urological tumours; however, it remains the most prevalent malignancy among males aged 15–40 years, with an age‐standardised incidence rate of 1.7 per 100 000 population according to Global Cancer Statistics 2022 (GLOBOCAN) [1, 2].

Notably, its incidence has been rising over recent decades, particularly in industrialised countries; however, diagnostic evaluation and treatments have remained almost unchanged [3].

The widespread implementation of scrotal ultrasonography (US), strengthened by advancements in high‐resolution imaging, has significantly increased the detection of testicular abnormalities. This has resulted in a notable rise in the identification of small testicular masses (STMs), including both palpable and non‐palpable lesions of uncertain malignant potential [4].

There is no universally accepted threshold size to define STMs. In this review, STMs are broadly defined as lesions measuring ≤20 mm, consistent with most available literature, while acknowledging that some guidelines and expert consensus statements adopt a 10‐mm threshold. The range between 10 mm and 20 mm therefore remains a grey area in clinical decision‐making [5, 6].

Several studies reported that more than 80% of STMs <20 mm that are excised have a benign histology [7, 8].

Radical orchidectomy (RO) is the standard of care for treating suspicious testicular masses when the contralateral testis is normal [9]. While RO is generally effective in ensuring complete removal of potentially malignant tissue, it carries substantial implications for patients. The loss of a testicle can adversely affect fertility and endocrine function, as well as diminish sexual satisfaction, contributing to psychological burdens such as anxiety and depression and negative body image [10].

These consequences have led to an increased interest in testis‐sparing surgery (TSS) as an alternative option. However, TSS is not yet widely practised, partly due to the limited availability of high‐quality randomised studies validating its efficacy and safety, which leaves the evidence base relatively sparse. International guidelines have acknowledged TSS as a potential option; however, there remains considerable variability in the diagnostic and therapeutic recommendations for STMs.

Therefore, to improve recommendations for clinicians treating patients with STMs, the aim of this study was to review the guidelines available on the diagnosis, treatment and follow‐up of STMs and to determine areas of consensus and discrepancy among them.

## Methods

A systematic search of PubMed, EMBASE, Scopus, Google Scholar, and the Cochrane Library was conducted to identify guidelines on the management of STMs and TSS published up to 1 December 2024. The search terms included ‘small testicular masses’, ‘testis‐sparing surgery’, ‘testicular surveillance’, ‘management’ and ‘treatment’, with filters for ‘review’, ‘systematic review’ and ‘meta‐analysis’ applied in PubMed. Only guidelines available in English were considered for inclusion. To ensure comprehensive coverage, manual searches were also performed of the websites of major international and national societies, as well as the official webpages of prominent urological and oncological organisations. Additionally, reference lists from relevant papers and guidelines were reviewed. Available materials were excluded if they did not adhere to a clinical guidelines framework or did not provide a comprehensive patient management strategy (e.g. focusing exclusively on diagnostic imaging). We approached this review with qualitative comparison of the content and of the strength of the recommendations of the guidelines based on three different steps in STM management: diagnostic evaluation, treatment, and follow‐up.

The quality of these guidelines was assessed using the Appraisal of Guidelines for Research and Evaluation II (AGREE II) tool by two authors (M.T. and G.F.; Table S1).

The levels of evidence were classified according to the criteria reported in Table S2.

## Results

The latest editions of five major guidelines were identified and analysed for their recommendations on the management of STMs and TSS. The European Association of Urology (EAU) Guidelines 2025 on TC, under section 5.4, provide specific recommendations regarding TSS [9]. The National Comprehensive Cancer Network (NCCN) Guidelines 2024 on TC present a schematic flowchart for STM management [11]. The AUA Guidelines, titled ‘Diagnosis and Treatment of Early‐Stage TC: AUA Guideline’, were initially published in 2019 and updated in 2023 [7]. These guidelines address multiple aspects of TC management, including detailed recommendations for TSS. The Canadian Urological Association (CUA) Consensus Guidelines 2022, ‘Management of Testicular Germ Cell Cancer’, provide an evidence‐based framework for STM management, including considerations for testis‐sparing procedures [12]. Finally, the European Society for Medical Oncology (ESMO) 2018 Consensus Conference on Testicular Germ Cell Cancer was convened to develop detailed recommendations on specific aspects of TC that were not comprehensively covered in the preceding ESMO Clinical Practice Guidelines [13].

Recommendations from the *Deutsche Gesellschaft für Urologie* (DGU) [14] and the *Association Française d'Urologie* (AFU) [15] largely overlap with those of the EAU Guidelines; therefore, they were not included in our review.

### Diagnosis and Staging

The reviewed guidelines consistently recommend US as the primary imaging modality for STMs, given its accessibility, high diagnostic accuracy, and non‐invasive nature. All major guidelines recognise US as the cornerstone for STM diagnosis, with strong recommendations and good level of evidence (Table 1). The EAU further supports the use of MRI in cases where US findings are inconclusive or to distinguish paratesticular from intratesticular lesions, particularly when TSS is being considered. Conversely, the AUA discourages the routine use of MRI for initial neoplastic suspicion, citing limited evidence supporting its necessity in standard diagnostic pathways.

**Table 1:** Summary of guidelines recommendations on diagnosis and staging of small testicular masses.

	EAU 2025	AUA 2023	NCCN 2024	CUA 2022	ESMO 2018
US	Always recommended (Strong recommendation)	Strong recommendation (Grade B)	Recommended (2A)	Always recommended.	Recommended (5A)
MRI	Recommended when US is inconclusive or to evaluate TSS cases	Not recommended for initial evaluation (Moderate; Grade C)	Not mentioned for local disease	Not mentioned for local disease	Helpful to distinguish intra‐ and extratesticular lesions
mpUS	Not mentioned	Not mentioned	Not mentioned	Not mentioned	CEUS helpful in characterising STMs <1 cm
Staging with CECT (chest/abdomen/pelvis)	Strong recommendation; deferred until malignancy confirmation	Strong recommendation; deferred until malignancy confirmation Abdomen and pelvis CECT (Strong Recommendation; Grade B). Chest CECT (Clinical Principle)	Recommended (not specified before or after pathology examination)	Recommended (not specified before or after pathology examination)	Staging imaging pre‐orchidectomy recommended (3A)

CECT, contrast‐enhanced CT; CEUS, contrast‐enhanced ultrasonography; CUA, Canadian Urological Association; EAU, European Association of Urology; ESMO, European Society for Medical Oncology; mpUS, multiparametric ultrasonography; NCCN, National Comprehensive Cancer Network; TSS, testis‐sparing surgery; US, ultrasonography.

With regards to advanced imaging techniques such as multiparametric ultrasonography (mpUS), which combines grey‐scale ultrasonography, colour‐Doppler ultrasonography (CDUS), contrast‐enhanced ultrasonography (CEUS) and sonoelastography (SE), despite some weak evidence in the literature, most of the reviewed guidelines do not incorporate these tools [16, 17]. The ESMO guidelines acknowledge the potential utility of CEUS, particularly for characterising STMs (<1 cm), suggesting a possible role in cases where conventional imaging provides limited diagnostic information.

The international guidelines generally do not provide specific recommendations regarding the size of testicular masses that would warrant surveillance, whether in terms of maximum length, volume, or the relationship between the lesion and the testicle, nor do they address the growth rate of the lesion. While there is no universally accepted threshold to define STMs, the AUA guidelines specifically define STMs as those ≤20 mm, whereas other guidelines do not establish a clear size cut‐off [7, 8]. Studies indicate that more than 80% of these STMs are histologically benign, but the lack of standardised criteria for determining which lesions are suitable for surveillance leaves room for clinical judgement, especially when considering factors such as lesion growth rate or ultrasonographic features [8].

A retrospective study conducted between January 2010 and December 2020 in 307 men with a STMs ≤20 mm concluded that STMs can be stratified and managed based on lesion size and US features [18]. The overall malignancy rate in men with an STM was 23.8%, with initial surveillance recommended for lesions <10 mm.

In another large study evaluating infertile couples (which included 4088 men), nearly 3% of those undergoing scrotal US for infertility evaluation were diagnosed with STMs [19]. While most incidental STMs did not show significant growth, patients with lesions >5 mm with vascularity were more likely to undergo surgery and potentially harbour malignancy, making surgical intervention necessary in these cases.

In line with this, the Delphi consensus panel suggested a 10‐mm diameter as the size cut‐off to define STMs [6]. This conclusion was based on previous findings, primarily from the previously cited large study in 307 men with an STM of 20 mm, which reported malignancy rates of 8.6% for lesions <10 mm, 46.3% for those measuring 10–14 mm, and 59.5% for those measuring 15–20 mm [18].

Regarding systemic staging, most guidelines recommend contrast‐enhanced CT (CECT) of the chest, abdomen and pelvis, after histopathological confirmation of malignancy. Both the EAU and AUA guidelines recommend deferring staging imaging until malignancy is confirmed, aiming to minimise unnecessary radiation exposure, and aligning with patient‐centred care principles. In contrast, the ESMO guidelines advocate for preoperative staging imaging in all patients undergoing surgery, irrespective of the histopathological findings.

### Management

Each case of STMs should ideally be evaluated by a multidisciplinary team with expertise in managing testicular tumours [9]. Since there is no standardised approach, management should be individualised based on clinical risk factors, tumour characteristics, and patient‐specific considerations. Options include active surveillance (AS), RO or TSS [6]. Key factors influencing the decision‐making process include the presence of unilateral or bilateral STMs, fertility status, gonadal function, tumour marker levels, CDUS findings, condition of the contralateral testis and, of course, shared decision‐making with the informed patient [18]. Preserving testicular function is a priority, particularly for patients with a history of infertility, hypogonadism, or an atrophic or absent contralateral testicle, while ensuring oncological safety remains paramount. Postoperative management should also involve a multidisciplinary team, with treatment decisions based on histopathological findings [9]. In cases of malignancy, factors such as tumour subtype, presence of risk features, gonadal function, fertility status and contralateral testis status will guide further management. Depending on these variables, the approach may include AS, adjuvant therapy, delayed RO, or radiotherapy [7, 9]. Patients should be informed that if TSS confirms TC, the risk of recurrence has been reported to be up to 26.9% [20, 21]. For patients with benign histology, postoperative evaluation of gonadal function is essential to address any endocrine abnormalities.

The psychological well‐being of patients undergoing orchidectomy should not be underestimated, as the loss of a testicle can significantly affect self‐image and emotional health. As studied by Skoogh et al. [22], the psychological impact of orchidectomy in TC survivors is a relevant yet often underestimated issue. Their findings suggest that many patients experience a sense of loss, decreased self‐esteem, and altered body image, particularly younger and single individuals. The absence of a testicle may further contribute to emotional distress, although its influence varies among patients. While psychological adaptation generally occurs over time, a subset of individuals continues to experience long‐term anxiety and depressive symptoms [22]. While loss of a testicle may cause anxiety or fear of recurrence, data on psychological outcomes after TSS are scarce, with few studies specifically addressing this aspect [23, 24].

### Treatment

The reviewed guidelines consistently highlight TSS as a key therapeutic option for STMs under specific conditions. The ESMO guidelines did not give any recommendation on surgical management of STMs.

The EAU, AUA, NCCN and CUA guidelines all recommend TSS for patients with small or indeterminate testicular masses, negative tumour markers, and a healthy contralateral testis (Table 2).

**Table 2:** Summary of guidelines recommendations on therapeutic management of small testicular masses.

	EAU 2025	AUA 2023	NCCN 2024	CUA 2022	ESMO
Synchronous bilateral tumours or in tumour in solitary testis	TSS when feasible, may be considered	Conditional recommendation (Grade C)	Indications for TSS	Partial orchidectomy by an experienced surgeon may be an alternative procedure to orchidectomy in very selected patients	Not mentioned TSS
Suspected interstitial cell or benign testicular tumours	TSS is a valid treatment option in patients with small or indeterminate testicular masses, negative tumour markers and a normal contralateral testis to avoid over‐treatment	An alternative to radical inguinal orchidectomy in highly selected patients wishing to preserve gonadal function with masses <2 cm, equivocal US findings and negative tumour markers (β‐hCG and AFP). (Conditional Recommendation; Grade C)	Not specified when it is a valid option	TSS should be performed prior to definitive orchidectomy in an experienced centre	Not mentioned TSS
Additional testicular biopsies	At least two additional testicular biopsies should be taken to exclude GCNIS	Multiple biopsies of the ipsilateral testicle normal parenchyma should be obtained for evaluation by an experienced pathologist. (Moderate Recommendation; Grade C)	Not mentioned	Mentioned but not specified how many	Not mentioned TSS
FSE	Strong recommendation	Always recommended	Always recommended	Always recommended	Not mentioned TSS
If discordance between FSE and final pathology	Delayed orchidectomy may be required	Not mentioned	Not mentioned	Not mentioned	Not mentioned TSS

AFP, alpha‐fetoprotein; CUA, Canadian Urological Association; EAU, European Association of Urology; ESMO, European Society for Medical Oncology; FSE, frozen section examination; GCNIS, germ cell neoplasia *in situ*; NCCN, National Comprehensive Cancer Network; TSS, testis‐sparing surgery; US, ultrasonography.

In the specific cases of synchronous bilateral testicular tumours or a tumour in a solitary testis, TSS is recommended when feasible, by the EAU, NCCN, AUA and CUA guidelines, with a primary focus on preserving hormonal function and fertility.

Detecting non‐palpable lesions can be difficult, making intra‐operative US a valuable tool to enhance identification. Depending on the case, US may be used alone or in combination with needle localisation to improve accuracy and facilitate precise surgical management [18].

Both the EAU and AUA recommend obtaining multiple testicular biopsies during TSS to exclude concomitant germ cell neoplasia *in situ* (GCNIS). The EAU guidelines specifically advise obtaining at least two biopsies from different regions of the ipsilateral testis to ensure comprehensive sampling. The AUA guidelines also support the use of additional biopsies, although they do not specify the required number, instead recommending that biopsies should be assessed by an experienced pathologist.

In addition, frozen section examination (FSE) is strongly endorsed across all guidelines addressing TSS. FSE serves as a critical intra‐operative diagnostic tool, with all guidelines agreeing on recommending RO if malignant histology emerges from FSE after TSS.

#### Surveillance of STMs

Although no major guidelines currently endorse surveillance of STMs, AS has emerged as a potential management strategy, particularly for incidentally detected lesions measuring <10 mm [25]. This approach requires systematic monitoring through physical examinations, scrotal US, and tumour marker assessments, aiming to minimise overtreatment while ensuring timely detection of malignant transformation. Despite its potential, only a limited number of studies have addressed this topic. A non‐systematic review by Niemczyk et al. [25] highlighted AS as a reasonable conservative strategy for managing STMs in carefully selected patients. The systematic review reported that the transition to active treatment ranged from 0% to 8%, with no compromise in oncological outcomes for STMs without risk factors for TC, with no signs of disseminated disease, negative tumour markers, and non‐palpable lesions not exceeding 10 mm [25].

Similarly, the Delphi consensus reached 100% agreement that, in the absence of clinical risk factors, an indeterminate STM in a solitary testis or bilateral testes could be managed with US surveillance [6]. The same applies to men with a non‐functioning or atrophic contralateral testis or signs of impaired gonadal function.

The decision to transition from AS to definitive treatment in cases of suspected TC is multifactorial and should be guided by both objective clinical criteria and patient‐related factors. According to the existing literature, key determinants include a progressive increase in lesion size, new or worsening US abnormalities (e.g. increased vascularity, irregular margins, heterogeneous echotexture), and serum tumour marker dynamics, if applicable [6, 18]. Additionally, patient anxiety and preference significantly influence the decision‐making process, as psychological distress related to uncertainty can impact adherence to surveillance protocols [9]. A structured follow‐up strategy, incorporating both imaging and shared decision‐making, is therefore essential to optimise patient outcomes while minimising unnecessary interventions.

The Delphi consensus recommends that the first follow‐up US should be performed at least 6 weeks after the initial scan. The AUA guidelines also support repeating US after 6–8 weeks if initial findings are inconclusive and tumour markers remain within normal limits [7]. However, further high‐quality research, particularly incorporating advanced imaging techniques such as mpUS and MRI, is needed to refine patient selection and optimise surveillance protocols.

To conclude, although emerging evidence and expert consensus support AS in highly selected cases, this approach remains investigational and is not formally endorsed by any of the major international guidelines.

#### Follow‐up after TSS

Follow‐up protocols after TSS for STMs are essential to ensure safe and favourable long‐term outcomes. Their primary goal is the early detection of local recurrence while preserving testicular function. However, surveillance strategies are not uniformly defined across the reviewed guidelines, as summarised in Table 3.

**Table 3:** Summary of guidelines recommendations on follow‐up of small testicular masses.

EAU 2025	AUA 2023	NCCN 2024	CUA 2022	ESMO
TSS has implications for ongoing surveillance of the testis	Due to high rates of local relapse, close monitoring with physical examination and ultrasonography of the testis are imperative	Specific follow‐up not mentioned	Specific follow‐up not mentioned	TSS not mentioned

CUA, Canadian Urological Association; EAU, European Association of Urology: ESMO, European Society for Medical Oncology; NCCN, National Comprehensive Cancer Network; TSS, testis‐sparing surgery.

Close monitoring helps mitigate the risk of missed residual disease or progression. The AUA particularly emphasises the need for rigorous follow‐up due to the high risk of local recurrence, recommending regular physical examinations and scrotal US. While these are critical for the early detection of suspicious changes, the guidelines do not specify exact timing. Periodic assessment of serum tumour markers is also a common clinical practice, although not explicitly addressed in the guidelines.

The EAU guidelines stress that patients undergoing TSS require close and prolonged follow‐up, especially when FSE and final pathology findings are discordant, to promptly detect recurrence or progression. Notably, only the EAU explicitly recommends delayed orchidectomy in cases where FSE initially suggests a benign lesion but the final pathology reveals malignancy, whereas other guidelines do not clearly state this recommendation.

In case of malignancy the management approach may vary and could involve AS in very selected patients, adjuvant treatment, delayed RO, or radiotherapy [6]. Patients should be made aware that if TSS confirms TC, the risk of recurrence can be as high as 26.9% [20, 21]. For those with benign histology, it is imperative to assess gonadal function postoperatively to identify and manage any endocrine issues.

If the FSE reveals GCNIS, a completion orchidectomy or radiotherapy may be considered, especially if adjacent biopsies also show GCNIS [26]. This is because GCNIS has a significant risk of progressing to cancer, with up to 50% of cases developing into invasive cancer within 5 years [26]. Chemotherapy is generally not recommended due to its limited efficacy in treating GCNIS [27]. For completely excised lesions, or in patients with a solitary testis, non‐functioning contralateral testis, or infertility, delayed radiotherapy with US surveillance may be an option [9].

Additionally, ≈2%–5% of patients with unilateral TC also have GCNIS in the contralateral testis, therefore, contralateral biopsy should be considered, particularly in the presence of risk factors such as a testis size <12 mL or an undescended testis. According to the EAU guidelines, contralateral biopsy is not necessary for patients over 40 years old without these risk factors [9].

To summarise, although follow‐up schedules vary among international guidelines, most agree on a combined strategy including regular physical examination, scrotal ultrasonography, and serum tumour marker assessment (alpha‐fetoprotein, β‐hCG and lactate dehydrogenase). The first ultrasonography scan is typically recommended within 6–8 weeks after surgery or initial diagnosis, followed by 3‐ to 6‐month intervals during the first year, and annual follow‐up thereafter depending on histology and clinical evolution. Long‐term evaluation of gonadal function is also encouraged, particularly in men with a solitary testis or impaired endocrine status. This integrated clinical, radiological, and biochemical approach supports early detection of recurrence while preserving testicular function.

#### Follow‐up after RO

If malignancy is confirmed after RO, staging and risk assessment should be completed with CECT (if not already performed) and repeat tumour markers. Treatment should follow established international guidelines, considering tumour type (seminoma or non‐seminomatous germ cell tumour), stage, prognostic classification, and individual factors such as fertility status, solitary testis, or non‐functioning contralateral testis [7, 9, 13]. Surveillance may be an option for low‐risk clinical stage I cases to preserve gonadal function. Since adjuvant chemoradiotherapy can impair testosterone and sperm production, patients should be counselled on potential endocrine and reproductive consequences, with testosterone replacement therapy initiated if necessary. Given that TC can already affect semen quality before RO, sperm cryopreservation or oncological testicular sperm extraction (oncoTESE) is strongly recommended for young men desiring fertility preservation [28, 29].

As mentioned earlier, ≈2%–5% of patients with unilateral TC also have GCNIS in the contralateral testis. Therefore, contralateral biopsy should be considered, especially when risk factors such as a testis size of <12 mL or undescended testis are present. According to the EAU guidelines, contralateral biopsy is not recommended for patients over 40 years old without these risk factors [9]. Moreover, infertile men undergoing RO, with contralateral testicular microcalcifications are at an increased risk of TC, therefore, contralateral biopsies should also be considered in these individuals [30].

### Summary of Key Consensus and Controversies

Overall, the reviewed international guidelines converge on several key points. US is consistently endorsed as the first‐line imaging modality for evaluating STMs, while MRI serves as a problem‐solving tool in selected cases. TSS is recognised as a feasible and safe option for indeterminate lesions with negative tumour markers, particularly in patients with a solitary testis or with bilateral tumours, provided that intra‐operative FSE is available.

Despite these shared recommendations, several areas of divergence remain. The size threshold defining STMs (10 mm vs 20 mm), the role of AS for small, incidentally detected lesions, the extent of intra‐operative biopsies to detect GCNIS, and the lack of standardised follow‐up protocols after TSS or RO represent ongoing controversies. These variations highlight the need for further evidence and international consensus to harmonise clinical practice.

## Discussion

The management of STMs and the application of TSS require a comprehensive, patient‐centred approach, starting with thorough counselling. All reviewed guidelines emphasise the importance of informing patients about all available therapeutic options, including the risks and benefits of TSS vs RO. This discussion should address the oncological safety, functional outcomes, and potential complications associated with each approach, ensuring that patients can make well‐informed decisions.

The choice of treatment pathway is particularly critical for patients undergoing TSS, as this procedure is indicated only in selected cases. These include patients with small or indeterminate testicular masses, normal tumour markers, and a healthy contralateral testis or patients with bilateral indeterminate testicular lesions or with STMs and a solitary testis and negative tumour markers.

In this setting, it is important to assess and address fertility preservation early. As described by Moody et al., 6%–24% of patients with TC are azoospermic at diagnosis, while 50% present with oligozoospermia [31]. Semen cryopreservation remains the preferred method for fertility preservation; however, it is underutilised, with only 24% of patients banking sperm [31]. Microdissection testicular sperm extraction (microTESE) at the time of orchidectomy (onco‐microTESE) is an effective option for azoospermic or severely oligozoospermic patients at diagnosis. Moreover, microTESE may still retrieve sperm in azoospermic patients after chemotherapy, highlighting its role in fertility preservation [28].

In these scenarios, TSS provides a balance between oncological control and the preservation of gonadal function. However, despite clear indications, significant variability persists across guidelines regarding the diagnostic pathways, the implementation of advanced imaging techniques for diagnosis, the surgical and follow‐up strategies.

A systematic review and meta‐analysis by Ager et al. [32] identified several US features as potential predictors of malignancy. Lesion size played a significant role, with masses ≤0.5 cm demonstrating a lower malignancy risk compared to those >0.6 cm, while no significant difference was found between 0.6–1.0‐cm lesions and 1.1–1.5‐cm lesions. Heterogeneous masses were associated with lower odds of malignancy compared to homogeneous masses. Additionally, echogenicity appeared to be a relevant factor, as hyperechoic masses had a lower malignancy risk compared to both hypoechoic and isoechoic lesions [32]. These findings highlight the importance of US characteristics in risk stratification and clinical decision‐making.

While US remains the ‘gold standard’ for STM evaluation, advanced imaging modalities such as MRI and mpUS may further enhance diagnostic precision. MRI, in particular, serves as a valuable problem‐solving tool in cases where US findings are equivocal or inconsistent with clinical presentations [33]. By providing detailed information on lesion morphology, functional characteristics, and tissue composition, MRI is especially effective in differentiating Leydig cell tumours (LCTs) from other testicular neoplasms [34]. A prospective study by Manganaro et al. [35] involving 44 consecutive patients with non‐palpable testicular lesions found scrotal MRI had a sensitivity of 89.47% and specificity of 95.65% for diagnosing LCTs. For malignant lesions, sensitivity was 95.65%, with 80.95% specificity. LCTs exhibited distinctive MRI features, aiding in the differential diagnosis of incidental testicular lesions [35].

These advanced imaging features hold promise for improving diagnostic accuracy and surgical planning, supporting conservative management or AS in selected patients.

Similarly, mpUS potentially offers significant diagnostic accuracy improvements by utilising tools such as CEUS and SE to differentiate between benign and malignant lesions [36]. A study by Goddi et al. [37] found that SE had a sensitivity of 87.5%, a specificity of 98.2%, a positive predictive value of 93.3%, a negative predictive value of 96.4%, and accuracy of 95.8% in distinguishing between malignancies and benign lesions. In another study, Aigner et al. observed that all neoplasms were identified as hard (blue) on the colorimetric map, with a sensitivity of 100%, a specificity of 81%, a negative predictive value of 100%, a positive predictive value of 92%, and accuracy of 94% [38]. Additionally, Pozza et al. reported that SE had a sensitivity of 81.8% and a specificity of 79.1% in identifying malignancies, while it demonstrated 100% specificity in discriminating non‐neoplastic lesions [39].

Despite the promising results, however, mpUS has not been incorporated into current international guidelines due to limited evidence supporting its routine use, the need for high expertise, and its availability in only a few centres. The Delphi consensus panel also does not recommend the routine use of mpUS for evaluating STMs, unless a CDUS is equivocal, and the modality is available and performed by experienced radiologists [6]. Further research is needed to validate its clinical utility and establish standardised protocols for its application.

While both mpUS and MRI show considerable promise in improving diagnostic accuracy, these techniques remain investigational and are not currently incorporated into international guidelines.

In future guideline iterations, mpUS and MRI could be incorporated as complementary tools for selected cases in specialised centres with appropriate expertise. Their role would likely focus on problem‐solving scenarios, such as indeterminate ultrasonography findings or candidates for TSS rather than as routine imaging modalities. Wider adoption would depend on further validation, cost‐effectiveness analyses, and the establishment of standardised acquisition and interpretation protocols.

Testicular biopsies during TSS remain a controversial topic. Although both EAU and AUA guidelines recommend biopsies to identify GCNIS, some experts question their necessity. This scepticism stems from the hypothesis that TC originates from the preneoplastic GCNIS and that detecting GCNIS may not alter patient management, while potentially increasing the risk of unnecessary complications [40]. However, it is now recognised that some prepubertal tumours are not GCNIS‐driven, suggesting that biopsies of residual testicular tissue may provide valuable diagnostic information [6]. Furthermore, recent EAU and International Society of Urological Pathology guidelines recommend classifying testicular tumours as GCNIS‐derived or non‐GCNIS‐derived, which supports the role of residual testis biopsies in clinical practice [41].

The use of FSE during TSS remains a critical intra‐operative tool, allowing real‐time differentiation between benign and malignant masses. However, discordance between FSE and final pathology results continues to present challenges. According to the EAU guidelines, the discordance rate is low, as FSE, when performed by experienced professionals, demonstrates a sensitivity of 99%, a specificity of 96%, and positive and negative predictive values of 98% and 97%, respectively [42]. This suggests that false‐negative results are extremely rare. However, when there is discordance between FSE and final pathology, management strategies may include delayed orchidectomy, intensified surveillance, or radiotherapy, emphasising the importance of clinician expertise and a multidisciplinary approach to decision‐making [9].

The smaller STMs (<5 mm) can present challenges for histopathological assessment, particularly when FSE is conducted. This procedure may cause tissue artefacts, potentially affecting morphological evaluation and immunohistochemical analysis. As a result, the Delphi panel recommended performing excisional biopsy for STMs <5 mm, with delayed RO considered in appropriate cases if malignancy is confirmed [6].

From a surgical perspective, RO with division of the spermatic cord at the internal inguinal ring remains the standard approach for TC [9]. For STMs, the Delphi panel noted that clamping the spermatic cord (Chevassu manoeuvre) before testis delivery is optional, as its role in preventing lymphovascular spread remains controversial. A scrotal approach should be avoided due to the risk of local tumour seeding, although evidence suggests it does not impact distant recurrence or survival [43]. Alternatively, a subinguinal approach, which preserves the inguinal canal and minimises nerve injury, may provide comparable oncological outcomes to the traditional technique [6].

Emerging evidence suggests that circulating microRNAs (miRNAs) may serve as promising biomarkers to distinguish benign from malignant testicular lesions. Recent studies have shown that selected miRNA signatures can improve diagnostic accuracy and potentially complement conventional imaging and tumour markers in the evaluation of STMs [1]. However, these findings are preliminary, and further validation is required before routine incorporation into clinical guidelines [44].

The variability in follow‐up protocols across guidelines reflects the ongoing debate over optimal post‐TSS surveillance. The EAU and AUA advocate for regular monitoring with physical examinations, imaging (e.g. US), and serum tumour marker evaluations, which remain the basis of care. These measures are crucial for detecting recurrence or progression, particularly in patients with discordant FSE and final pathology results. However, the absence of detailed follow‐up protocols in the NCCN and CUA guidelines, and the lack of recommendations from ESMO, highlight the need for further research and standardisation in this domain.

In conclusion, the management of STMs and the implementation of TSS require a delicate balance between oncological safety and the preservation of testicular function. While significant progress has been made in the development of guidelines, gaps remain in the integration of advanced imaging modalities and standardised follow‐up protocols. Imaging modalities such as mpMRI and mpUS show great promise for enhancing diagnostic accuracy and surgical planning, but further studies are required to validate their routine clinical application.

Testis‐sparing surgery, supported by evidence‐based protocols, remains a viable option for selected patients. However, its success depends on meticulous preoperative evaluation, the use of intra‐operative tools such as FSE, and comprehensive follow‐up strategies to optimise outcomes and mitigate risks.

By adhering to guideline recommendations and maintaining a patient‐centred approach, clinicians can ensure a high standard of care while advancing the management of testicular masses.

Nevertheless, future research should prioritise prospective multicentre studies directly comparing TSS and RO to establish robust evidence on long‐term oncological control, endocrine and fertility outcomes, and quality of life. Moreover, health‐economic analyses assessing the cost‐effectiveness of advanced imaging modalities, such as mpUS and mpMRI, are warranted to guide resource allocation and optimise patient care.

## Funding

None.

## Disclosure of Interests

None declared.

## Author Contributions

Study conception and design: M.T, G.F, K.H.P. Acquisition of data: M.T, G.F, K.H.P. Analysis and interpretation of data: M.T, G.F, K.H.P. Drafting of the manuscript: M.T, G.F, K.H.P. A.M. Critical revision of the manuscript for important intellectual content: All authors. Statistical analysis: None. Obtaining funding: None. Supervision: G.F, K.H.P.

## Supporting information


**Table S1.** AGREE II quality assessment of included guidelines.

**Table S2.** Level of evidence.
