# Supplementary material for: Guideline of guidelines: management of small testicular masses
Source: BJU Int. 2025 Dec 24;137(3):444–53. doi: 10.1111/bju.70131 (PMC12907781; doi:10.1111/bju.70131)
Supplement: Supplementary file 1 — Table S1. AGREE II quality assessment of included guidelines. [file BJU-137-444-s002.docx]

Supplementary Table 1: AGREE II Quality Assessment of Included Guidelines

| **AGREE II** | EAU 2024 | | NCCN 2024 | | AUA 2023 | | CUA 2022 | | ESMO 2018 | |
| --- | --- | --- | --- | --- | --- | --- | --- | --- | --- | --- |
| **Domain 1 - Scope and Purpose** | | | | | | | | | | |
| The overall objective(s) of the guideline is (are) specifically described. | 7 | 7 | 7 | 6 | 7 | 7 | 6 | 6 | 6 | 6 |
| The health question(s) covered by the guideline is (are) specifically described. | 7 | 7 | 6 | 6 | 7 | 7 | 6 | 6 | 6 | 7 |
| The population (patients, public, etc.) to whom the guideline is meant to apply is specifically described. | 6 | 7 | 6 | 6 | 6 | 6 | 6 | 6 | 6 | 6 |
| **Domain 2 - Stakeholder Involvement** | | | | | | | | | | |
| The guideline development group includes individuals from all relevant professional groups. | 7 | 6 | 7 | 6 | 6 | 6 | 6 | 6 | 7 | 7 |
| The views and preferences of the target population (patients, public, etc.) have been sought. | 6 | 5 | 5 | 5 | 6 | 6 | 5 | 6 | 6 | 6 |
| The target users of the guideline are clearly defined. | 7 | 6 | 6 | 6 | 6 | 7 | 6 | 5 | 6 | 7 |
| **Domain 3 - Rigour of Development** | | | | | | | | | | |
| Systematic methods were used to search for evidence. | 6 | 6 | 5 | 6 | 7 | 7 | 7 | 6 | 7 | 6 |
| The criteria for selecting the evidence are clearly described. | 7 | 7 | 6 | 5 | 7 | 6 | 6 | 5 | 7 | 6 |
| The strengths and limitations of the body of evidence are clearly described. | 7 | 6 | 6 | 6 | 7 | 7 | 5 | 5 | 7 | 6 |
| The methods for formulating the recommendations are clearly described. | 6 | 6 | 5 | 5 | 7 | 7 | 5 | 6 | 7 | 7 |
| The health benefits, side effects, and risks have been considered in formulating the recommendations. | 7 | 7 | 6 | 5 | 6 | 7 | 6 | 4 | 6 | 6 |
| There is an explicit link between the recommendations and the supporting evidence. | 6 | 7 | 5 | 6 | 6 | 6 | 4 | 5 | 7 | 7 |
| The guideline has been externally reviewed by experts prior to its publication. | 5 | 6 | 5 | 5 | 6 | 6 | 5 | 5 | 6 | 6 |
| A procedure for updating the guideline is provided. | 7 | 7 | 6 | 6 | 6 | 7 | 5 | 5 | 3 | 3 |
| **Domain 4 - Clarity of Presentation** | | | | | | | | | | |
| The recommendations are specific and unambiguous. | 6 | 6 | 5 | 5 | 6 | 6 | 6 | 5 | 5 | 5 |
| The different options for management of the condition or health issue are clearly presented. | 6 | 6 | 5 | 5 | 6 | 6 | 5 | 4 | 5 | 5 |
| Key recommendations are easily identifiable. | 5 | 6 | 4 | 5 | 6 | 6 | 5 | 5 | 5 | 5 |
| **Domain 5 – Applicability** | | | | | | | | | | |
| The guideline describes facilitators and barriers to its application. | 6 | 6 | 4 | 5 | 7 | 7 | 5 | 5 | 6 | 5 |
| The guideline provides advice and/or tools on how the recommendations can be put into practice. | 6 | 6 | 5 | 4 | 6 | 6 | 5 | 6 | 5 | 6 |
| The potential resource implications of applying the recommendations have been considered. | 5 | 6 | 4 | 5 | 5 | 5 | 6 | 5 | 5 | 5 |
| The guideline presents monitoring and/or auditing criteria. | 6 | 5 | 5 | 5 | 6 | 5 | 5 | 4 | 6 | 5 |
| **Domain 6 - Editorial Independence** | | | | | | | | | | |
| The views of the funding body have not influenced the content of the guideline. | 7 | 7 | 6 | 6 | 7 | 6 | 6 | 6 | 6 | 6 |
| Competing interests of guideline development group members have been recorded and addressed. | 6 | 7 | 6 | 6 | 6 | 7 | 6 | 6 | 6 | 6 |
| **Overall Assessment** | | | | | | | | | | |
| Rate the overall quality of this guideline. | 7 | 7 | 6 | 6 | 7 | 7 | 6 | 6 | 6 | 6 |
| I would recommend this guideline for use. | Yes | Yes | Yes | Yes | Yes | Yes | Yes | Yes | Yes | Yes |
