# Supplementary material for: Guideline of guidelines: management of small testicular masses
Source: BJU Int. 2025 Dec 24;137(3):444–53. doi: 10.1111/bju.70131 (PMC12907781; doi:10.1111/bju.70131)
Supplement: Supplementary file 2 — Table S2. Level of evidence. [file BJU-137-444-s001.docx]

Supplementary Table 2: level of evidence

| **Evidence grade** | **Evidence Strength A (High Certainty)** | **Evidence Strength B (Moderate Certainty)** | **Evidence Strength C (Low Certainty)** |
| --- | --- | --- | --- |
| **1: Strong**  **Recommendation (Net benefit or harm substantial)** | -Benefits > Risks/Burdens  (or vice versa)  -Net benefit (or net harm) is substantial  -Applies to most patients in most circumstances and future research is unlikely to change confidence | -Benefits > Risks/Burdens (or  vice versa)  -Net benefit (or net harm) is substantial  -Applies to most patients in most circumstances but better evidence could change confidence | -Benefits > Risks/Burdens (or  vice versa)  -Net benefit (or net harm) appears substantial  -Applies to most patients in most circumstances but better evidence is likely to change confidence (rarely used to support a Strong Recommendation) |
| **2: Moderate Recommendation (Net benefit or harm moderate)** | -Benefits > Risks/Burdens (or vice versa)  -Net benefit (or net harm) is moderate  -Applies to most patients in most circumstances and future research is unlikely to change confidence | -Benefits > Risks/Burdens (or vice versa)  -Net benefit (or net harm) is moderate  -Applies to most patients in most circumstances but better evidence could change confidence | -Benefits > Risks/Burdens (or vice versa)  -Net benefit (or net harm) appears moderate  -Applies to most patients in most circumstances but better evidence is likely to change confidence |
| **3: Conditional Recommendation (Net benefit or harm comparable to other options)** | -Benefits = Risks/Burdens  -Best action depends on individual patient circumstances  -Future Research is unlikely to change confidence | -Benefits = Risks/Burdens  -Best action appears to depend on individual patient circumstances  -Better evidence could change confidence | -Balance between Benefits & Risks/Burdens unclear  -Net benefit (or net harm) comparable to other options  -Alternative strategies may be equally reasonable  -Better evidence likely to change confidence |
| **4: Clinical Principle** | A statement about a component of clinical care that is widely agreed upon by urologists or other clinicians for which there may or may not be evidence in the medical literature. | | |
| **5: Expert Opinion** | A statement, achieved by consensus of the Panel, that is based on members' clinical training, experience, knowledge, and judgment for which there may or may not be evidence in the medical literature. | | |

*Adapted from the American Urological Association (AUA) Guidelines, 2023*
